# Bioavailability of a Lipidic Formulation of Curcumin in Healthy Human Volunteers 

**DOI:** 10.3390/pharmaceutics4040517

**Published:** 2012-10-09

**Authors:** Yogesh B. Pawar, Bhushan Munjal, Saurabh Arora, Manoj Karwa, Gunjan Kohli, Jyoti K. Paliwal, Arvind K. Bansal

**Affiliations:** 1 Department of Pharmaceutics, National Institute of Pharmaceutical Education and Research (NIPER), S.A.S. Nagar, Punjab-160062, India; Email: yogesh_cpn@yahoo.com (Y.B.P.); bhushanmunjal@gmail.com (B.M.); gunjan@niper.ac.in (G.K.); jyotip@niper.ac.in (J.K.P.); 2 Auriga Research Limited, Kirti Nagar Industrial Area, New Delhi-110015, India; Email: saurabh@aurigaresearch.com (S.A.); manojkarwa@aurigaresearch.com (M.K.)

**Keywords:** curcumin, bioavailability, absorption, pharmacokinetic modeling

## Abstract

Numerous publications have reported the significant pharmacodynamic activity of Curcumin (CRM) despite low or undetectable levels in plasma. The objective of the present study was to perform a detailed pharmacokinetic evaluation of CRM after the oral administration of a highly bioavailable lipidic formulation of CRM (CRM-LF) in human subjects. *C*_max_, *T*_max_ and AUC_0–∞_ were found to be 183.35 ± 37.54 ng/mL, 0.60 ± 0.05 h and 321.12 ± 25.55 ng/mL respectively, at a dose of 750 mg. The plasma profile clearly showed three distinct phases, *viz*., absorption, distribution and elimination. A close evaluation of the primary pharmacokinetic parameters provided valuable insight into the behavior of the CRM after absorption by CRM-LF. CRM-LF showed a lag time (*T*_lag_) of 0.18 h (around 12 min). Pharmacokinetic modeling revealed that CRM-LF followed a two-compartment model with first order absorption, lag time and first order elimination. A high absorption rate constant (*K*_01_, 4.51/h) signifies that CRM-LF ensured rapid absorption of the CRM into the central compartment. This was followed by the distribution of CRM from the central to peripheral compartment (*K*_12_, 2.69/h). The rate of CRM transfer from the peripheral to central compartment (*K*_21_, 0.15/h) was slow. This encourages higher tissue levels of CRM as compared with plasma levels. The study provides an explanation of the therapeutic efficacy of CRM, despite very low/undetectable levels in the plasma.

## 1. Introduction

In recent years, polyphenolic antioxidants have gained a lot of importance due to their potential as prophylactic and therapeutic agents for cancer, diabetes, cardiovascular diseases, autoimmune diseases, neurodegenerative disorders, aging and other diseases [1]. Recently, therapeutic utility of these polyphenolic compounds is linked with their ability to block amyloid formation, a common feature of many degenerative diseases including Alzheimer’s, type II diabetes, Parkinson’s, Creutzfield-Jacob’s and Huntington’s disease [2,3,4].

Curcumin (CRM), (1*E*,6*E*)-1,7-bis(4-hydroxy-3-methoxyphenyl)-1,6-heptadiene-3,5-dione or diferuloylmethane is the most active constituent of turmeric. It is acts on multiple target sites and is non-toxic even at high doses [5]. CRM is undergoing clinical trials for several human ailments like familial adenomatous polyposis, inflammatory bowel disease, ulcerative colitis, colon cancer, pancreatic cancer, hypercholesterolemia, atherosclerosis, pancreatitis, psoriasis, chronic anterior uveitis, arthritis, Crohn’s disease and neurological diseases [6]. Transformation of this “wonder molecule” into “drug” is severely hampered by its poor oral bioavailability. The poor aqueous solubility, chemical instability in alkaline medium [7] rapid metabolism [8] and poor membrane permeation [9] have been reported to contribute towards poor oral bioavailability of CRM.

In a phase I clinical study, CRM doses of between 0.45 g and 3.6 g daily were administered for a period up to four months. Efficacy of CRM was related to induction of glutathione *S*-transferase enzymes, inhibition of prostaglandin E2 (PGE_2_) production, or suppression of an oxidative DNA adduct (M_1_G) formation. There was a 62% decrease in inducible PGE_2_ production compared with predose levels, irrespective of the fact that negligible levels (around 10 ng/mL) of CRM, its glucuronide and sulfate metabolites were detected in plasma and urine [10]. The outcome of additional studies has intrigued scientists, wherein significant pharmacodynamic events have been observed despite undetectable levels in plasma [11,12]. These observations indicate that the plasma levels of CRM do not correlate well with its therapeutic efficacy. Thus there is a need to understand detailed pharmacokinetics of CRM in order to gain insight into its pharmacodynamic effectiveness. This understanding will help in optimizing oral delivery of CRM. 

Human pharmacokinetics of orally administered CRM has not been studied extensively, as naive CRM was not able to provide detectable plasma levels. Our research group is actively engaged in the oral delivery of CRM. We have screened various formulation strategies [13] and engendered a lipidic formulation of CRM (CRM-LF). CRM-LF consisted of CRM (6.17% *w*/*w*) Gelucire^®^ 44/14 (16.46% *w*/*w*), Labrasol (5.76% *w*/*w*), Vitamin E TPGS (3.29% *w*/*w*), PEG 400 (55.55% *w*/*w*), ethanol (8.23% *w*/*w*), anhydrous citric acid (2.88% *w*/*w*) and HPMC E5 (1.64% *w*/*w*). CRM-LF forms the nanosized globules upon dilution with aqueous medium. CRM-LF demonstrated *C*_max_ and AUC_(0–*t*)_ of 527.01 ng/mL and 2393.03 ng h/mL respectively, in comparison to 32.29 ng/mL and 38.07 ng h/mL respectively, achieved with naive CRM in male Sprague-Dawley rats. CRM-LF was also found to be more efficacious than naive CRM, in diabetic encephalopathy and diabetic neuropathy in male Sprague-Dawley rats (data communicated elsewhere). 

The objective of present study was to perform detailed pharmacokinetics of CRM after oral administration of CRM-LF, in human subjects. Additional information about the fate of CRM in the biological system was generated by performing pharmacokinetic modeling. 

## 2. Experimental Section

### 2.1. Materials

CRM was purchased from HiMedia Laboratories Pvt Ltd., Mumbai, India. Gelucire^®^ 44/14 and Labrasol were gift samples by Gattefosse Pvt. Ltd., Saint Priest Cedex, France. Vitamin E TPGS NF was gift sample from Isochem, Gennevilliers, France. PEG 400 was purchased from Merck, Mumbai, India. Absolute ethanol was purchased from Hong Yang Chemical Co. Ltd., Jiangsu, China. Hydroxylpropyl methyl-cellulose (HPMC E5) was purchased from Colorcon Asia Pvt. Ltd., Goa, India. Anhydrous citric acid was purchased from merck Ltd., Mumbai, India. All chromatographic solvents were purchased from Merck, Daermstadt, Germany. 

### 2.2. Methods

#### 2.2.1. Characterization of CRM-LF

##### 2.2.1.1. Analysis of CRM in CRM-LF

CRM content in CRM-LF was determined by validated reverse phase-HPLC assay method. Mobile phase comprising of acetonitrile and citric acid solution (pH 3.0) in proportion of 50:50 *v*/*v*, previously filtered through 0.45 µm filter and sonicated, was pumped isocratically at a flow rate of 1 mL/min. LiChrospher^®^ C_18_ analytical column (200 × 4.6 mm, 5 µm particles; Merck) was used as the stationary phase. Injection volume and run time were 20 µL and 8 min respectively. UV-Visible detector set at λ_max_ of 430 nm was used to monitor the eluate. 

##### 2.2.1.2. Globule Size Analysis of CRM-LF upon Dilution

Simulated gastric fluid was prepared by dissolving sodium chloride (100 mg) and pepsin (160 mg) in 25 mL of water, followed by addition of 0.35 mL of hydrochloric acid and making the volume up to 50 mL with water. CRM-LF was diluted 250 times in simulated gastric fluid that was previously filtered through a 0.45 μm filter. The droplet size was measured by dynamic light scattering (Nano ZS, Malvern, UK) by taking the average of five measurements. 

#### 2.2.2. Pharmacokinetic Study

##### 2.2.2.1. Demographic Data of Healthy Volunteers

Twelve healthy male subjects involved in a study had average age of 26.45 ± 5.02 years, average height of 164.09 ± 5.00 cm, average weight of 57 ± 5.91 kg. Subjects were admitted 24 h prior to the study drug administration, supervised for at least 10 h of overnight fasting, and confined until collecting the 24 h sample. 

##### 2.2.2.2. Study Design

An open label, balanced, randomized, parallel design study was performed to evaluate bioavailability of single oral dose of 750 mg of CRM. Protocol no. ARL-BE-021-CURC-2009-(Version 02) involving healthy human adult male subjects was approved by Hippocrates Independent Ethics Committee, New Delhi, India. Volunteers were instructed not to take CRM in their meals at least one week prior to the study. CRM and garlic free meals were provided to the volunteers during the study. Consumption of alcohol, tobacco, any beverages or foods containing methyl xanthines, e.g., caffeine (coffee, tea, cola, cocoa, chocolate, *etc*.) and grapefruit juice was prohibited for the subjects 48 hours prior to drug administration, until the collection of the last sample of the respective study period. No medicines, including over-the counter products, were allowed for 14 days preceding the study and for the entire duration of the study.

##### 2.2.2.3. Dosing and Blood Sampling

The subjects were dosed after an overnight fasting of 10 h. The fasting state was continued for 4 h post dose. Blood samples (4 mL) were collected in lithium-heparin vacutainers predose and 0.25, 0.50, 0.75, 1, 1.5, 1.50, 2, 3, 4, 6, 8, 12, 18, 24 h after dosing. Separated plasma after centrifugation, was stored at −20 °C until analyzed. 

##### 2.2.2.4. Sample Processing

Calibration curve standards, quality control samples and subject plasma samples were withdrawn from deep freezer, allowed to thaw at room temperature and vortexed. Five hundreds microliter sample was transferred to RIA vial and 25 μL of 10.5 μg/mL betamethasone (BMS) solution was added as internal standard, followed by vortexing for 15 s. Ethyl acetate (2.5 mL) was added and vortexed for 15 min. Contents were centrifuged at 5000 rpm at 4 °C for 5 min. Supernatant was removed and evaporated under nitrogen at 37 °C. The dried residue was reconstituted with 0.5 mL of methanol, transferred into HPLC vials and injected into LC-MS/MS system. 

##### 2.2.2.5. Analysis of CRM in Plasma by the LC-MS/MS Method

The plasma matrix for calibration curve standard and quality control samples was obtained from Prathma Blood Centre, Vasna, Ahmedabad, India. The matrix used for calibration curve standards and quality control samples was screened for interfering endogenous substances. There were no interfering peaks at the retention time of CRM. The LC-MS/MS system (API 3000 LC/MS/MS system, Software Analyst 1.4.2) consisted of PerkinElmer LC pump and PerkinElmer Auto Sampler. Hypurity Advance Column (50 × 4.6 mm, 5 μm) was used. Column oven temperature and injection volume were 35 °C and 10 μL respectively. Mobile phase consisting of 0.1% formic acid and acetonitrile (20:80 *v*/*v*) was pumped isocratically at a flow rate of 1 mL/min. Run time of method was 7 min and Peltier temperature was 4 °C. Method was validated for accuracy, precision, stability and dilution integrity. A total of nine calibration curves were generated with the study sample. The concentration range for CRM employed in the standard curve ranged from 2.12 ng/mL to 248.95 ng/mL. Calibration curves were constructed by weighted (1/*x*^2^) linear regression by calculating the peak area ratio of CRM:BMS. The co-efficient of correlation (*r*^2^) was greater than 0.99 for all calibration curves. 

##### 2.2.2.6. Data Analysis

All pharmacokinetic analysis was carried out by using WinNonlin Version 5.2 (Pharsight Corporation, Mountain View, California, USA).

##### 2.2.2.7. Compartmental Modeling

Log transformed plasma concentration time profile (log concentration *vs.* time) was plotted, as the first step of compartment modeling and visually evaluated. The profile was subsequently fitted to four models, *viz*. (i) one-compartment model with first order absorption, no lag time and first order elimination, (ii) one-compartment model with first order absorption, with lag time and first order elimination, (iii) two-compartment model with first order absorption, no lag time and first order elimination and (iv) two-compartment model with first order absorption, lag time and first order elimination. Models (ii) and (iv) were identical to model (i) and (iii) respectively, except that they had an additional calculated parameter of lag time *T*_lag_. 

Decision on the best fit compartment model for CRM-LF was based on the visual evaluation of predicted and observed plasma concentration profiles. The statistical parameters: corrected sum of squared observations (CSS), sum of squared residuals (SSR), standard error of the weighted residuals (S), correlation coefficient between the observed and predicted values (CORR_(OBS,PRED)), Akaike Information Criterion (AIC) and Schwarz Bayesian Criterion (SBC) were determined to enable the decision on the best fit model. 

## 3. Results and Discussion

### 3.1. Characterization of CRM-LF

#### 3.1.1. Analysis of CRM in CRM-LF

CRM content in CRM-LF formulation was 66.7 mg/mL, as determined by HPLC assay. This actual content of CRM in CRM-LF was found to be 99.5% of the added (theoretical) quantity of CRM. 

#### 3.1.2. Globule size Analysis of CRM-LF upon Dilution

**Figure 1:**
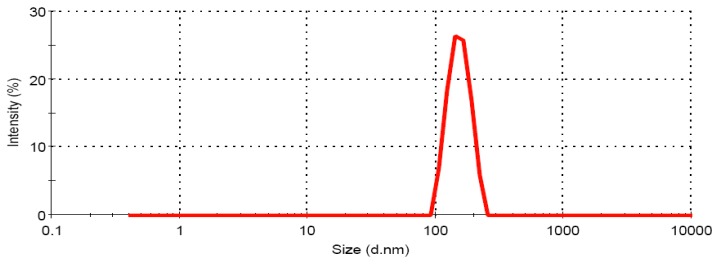
Zeta sizer analysis of a diluted lipidic formulation of Curcumin (CRM-LF).

Figure 1 shows the results for Zeta sizer analysis of diluted CRM-LF. Globule size of diluted CRM-LF was found to be 212.3 ± 18.9 nm with polydispersibility index of 0.253.

### 3.2. Bioanalytical Method Validation

**Figure 2:**
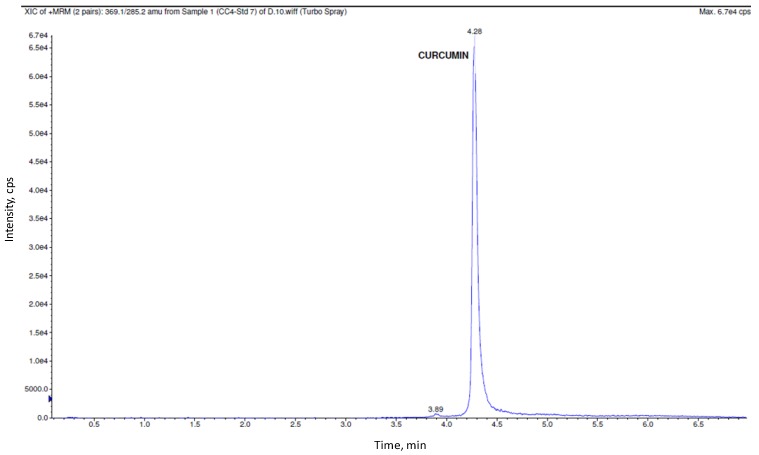
Representative chromatogram for calibration curve standard of CRM.

The precision for the quality control samples of CRM ranged from 7.4% to 10.3% and accuracy for the quality control samples ranged from 91.7% to 94.7%. Figure 2 shows representative chromatogram for calibration curve standard of CRM.

### 3.3. Pharmacokinetic Study

The pharmacokinetics of CRM in humans has been poorly understood because of its poor oral bioavailability that leads to undetectable plasma levels and makes the calculation of pharmacokinetic parameters difficult. In several studies, negligible or no plasma levels of CRM were observed in humans, even after oral doses of as high as 8 g. In 25 advanced pancreatic cancer patients, CRM was barely detectable when a dose of 8 g was administered daily for two months [14]. CRM was not detectable in blood and urine after administration of 440–2200 mg of curcuma extract (equivalent to 36–180 mg CRM) per day for 29 days, in colon adenocarcinoma patients [15]. In yet another study, out of 34 patients who consumed 10 and 12 g of CRM, only two patients showed detectable (around 50 ng/mL) plasma levels of CRM [16]. In another study, at a 2 g dose, Biocurcumax showed *C*_max_ and AUC_0–∞_ of 456.88 ng/mL and 3201.28 ng h/mL, which was at a three-fold and six-fold improvement in respective pharmacokinetic parameters as compared to equivalent dose of free CRM [17]. However the authors did not determine the pharmacokinetic parameters beyond *C*_max_ and AUC. In a recent study, administration of 650 mg curcuminoids to healthy volunteers led to undetectable plasma levels. Solid lipid CRM particle (SLCP) at a 650 mg dose (equivalent to 130 mg of CRM) from the trademark “Longvida” provided *C*_max_ of 22.43 ng/mL and CRM was detectable upto 8 h post dose [18].

**Figure 3:**
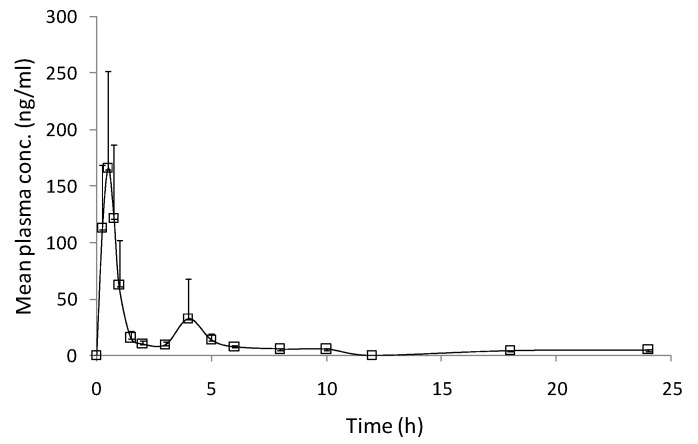
Mean plasma CRM concentration *vs*. time profile obtained after oral administration of CRM-LF to human volunteers at a dose of 750 mg.

Figure 3 shows mean plasma CRM concentration-time profile, obtained after oral administration of CRM-LF, while the pharmacokinetic parameters are given in Table 1. 

**Table 1:** Pharmacokinetic parameters of CRM after the administration of CRM-LF to human volunteers at a dose of 750 mg.

Pharmacokinetic parameters	Value (mean ± SEM)
*C*_max_ (ng/mL)	183.35 ± 37.54
*T*_max_ (h)	0.60 ± 0.05
t_1/2_ (h)	12.85 ± 4.57
AUC_0–*t*_ (ng.h/mL)	231.31 ± 24.45
AUC_0–∞_ (ng.h/mL)	321.12 ± 25.55

CRM-LF showed biphasic response with *C*_max_ of 183.35 ± 37.54 ng/mL, AUC_0–∞_ of 321.12 ± 25.55 ng h/mL, while *T*_max_ was observed at 0.60 ± 0.05 h. A higher degree of interindividual variability in *C*_max_ and half-life was observed in present pilot study. To address this issue of variability a study with a larger number of volunteers is needed. 

CRM, being a polyphenolic compound is prone to glucuronide formation and hence might undergo enterohepatic recirculation, possibly contributing to observed biphasic response [13]. 

Enhanced oral bioavailability of CRM with CRM-LF can be attributed to spontaneous emulsification into globules of nano-size range. These fine globules are rapidly absorbed into systemic circulation. Additionally encapsulation of CRM in globules might shield it from pH mediated chemical degradation, thus improving the fraction of CRM to be absorbed. 

Lipidic formulations enhance drug absorption by a number of ancillary mechanisms, including drug solubilisation, inhibition of *P*-glycoprotein-mediated drug efflux and preabsorptive metabolism by gut membrane-bound cytochrome enzymes and increasing gastrointestinal membrane permeability. 

The role of formulation excipients in enhanced oral bioavailability was investigated using Caco-2 cell culture based transport studies. The *P*_app_ value of naive CRM, CRM-LF and naïve CRM along with individual excipient was determined. CRM-LF showed statistically significant enhancement in *P*_app_ value (21.23 × 10^−6^ cm/s), when compared to naive CRM (3.34 × 10^−6^ cm/s). Further, the Labrasol present in CRM-LF contributed the maximum, amongst various excipients towards enhanced permeation. Additional studies on monolayer integrity of Caco-2 cell monolayers, which involved transepithelial electrical resistance (TEER) values and scanning electron microscopy analysis before and after transport studies, showed membrane disruption to be contributor in enhanced oral bioavailability of CRM [19]. 

The contribution of lipid digestion towards enhanced oral bioavailability was also investigated in male SD rats by incorporating orlistat (1% *w*/*w*) in CRM-LF (CRM-LF-OLT). Orlistat, being lipase inhibitor blocked the chylomicron formation and subsequently bioavailability was reduced, highlighting the importance of lipid digestion in oral bioavailability enhancement [20]. 

### 3.4. Compartmental Modeling

Quantifiable plasma levels were obtained upon oral administration of CRM-LF up to 24 h. This allowed for compartment modeling to establish the best-fit model that can help in designing drug delivery system for oral delivery of CRM. 

**Figure 4:**
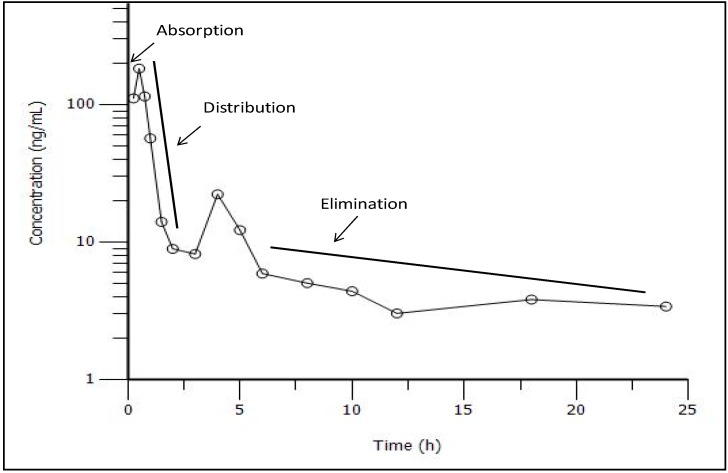
Log transformed plasma concentration profile of CRM-LF showing the absorption phase and rapid distribution phase followed by the slower elimination phase.

The plasma profile in Figure 4 clearly showed three distinct phases, *viz.*, absorption, distribution and elimination. This indicated that the pharmacokinetic profile could be fitted to a two-compartment model. 

**Figure 5:**
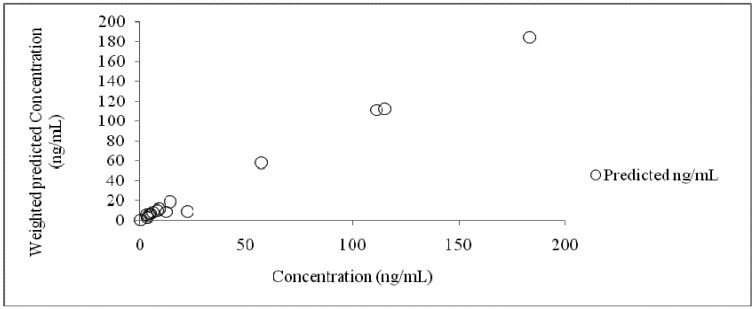
Scatter plot showing relation of weighted predicted plasma concentration *versus* the observed plasma concentration at 750 mg dose.

Based on visual evaluation, a two-compartment model with a first order absorption, lag time and first order elimination was considered the best fit. This model showed the smallest SSR, AIC, SBC and the highest correlation between the observed and predicted values in comparison with other three models. The scatter plots showing the correlation of observed and predicted values and the residuals are shown in Figure 5.

A nearly perfect correlation was obtained with this model having the correlation coefficient 0.9972.Predicted plasma concentrations for each time point, along with deviation from the observed concentrations as residuals, are summarized in Table 2. 

**Figure 6:**
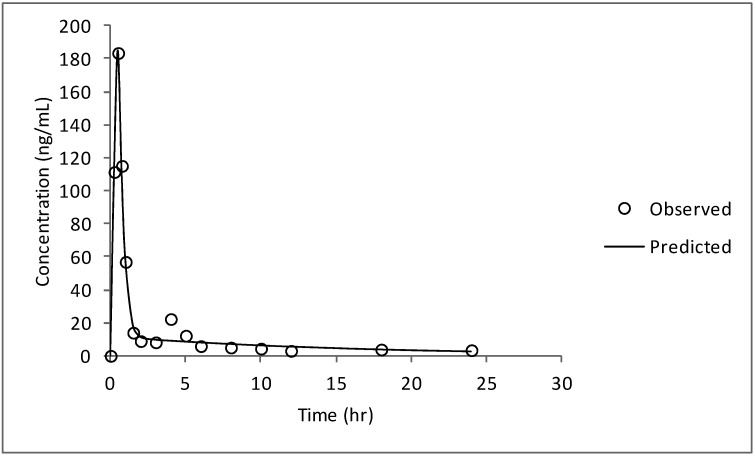
Plot of predicted and observed plasma concentration *vs.* time profile for CRM-LF for two-compartment model with lag time.

The predicted plasma concentration *vs.* time profile is plotted as a line, along with the observed concentrations as circles in Figure 6. It can be seen from the residuals and the graph that this model was able to predict the plasma concentration profile quite accurately for CRM-LF.

**Table 2:** Observed, predicted plasma concentrations and residuals for a two-compartment model with lag time.

Time (h)	Concentration (ng/mL)	Predicted (ng/mL)	Residual (ng/mL)
0	0	0	0
0.25	111.42	111.34	0.07
0.5	183.51	184.81	−1.30
0.75	115.13	112.37	2.75
1	56.92	58.14	−1.22
1.5	14.04	18.58	−4.54
2	8.94	11.50	−2.56
3	8.19	9.77	−1.58
4	22.29	9.16	13.12
5	12.23	8.61	3.61
6	5.9	8.09	−2.19
8	5.03	7.15	−2.12
10	4.39	6.31	−1.92
12	3.03	5.57	−2.54
18	3.82	3.84	−0.02
24	3.4	2.64	0.75

The primary pharmacokinetic parameters such as volume of distribution (*V*_D1_), absorption rate constant (*K*_01_), elimination rate constant (*K*_10_), distribution rate constant from central to peripheral compartment (*K*_12_), distribution rate constant from peripheral to central compartment (*K*_21_) and lag time (*T*_lag_) were calculated and are summarized in Table 3. 

**Table 3:** Pharmacokinetic parameters calculated using the best fit model.

Parameter	Units	Estimate
*Absorption phase*		
*C*_max_	ng/mL	195.87
*T*_max_	h	0.41
AUC	h.ng/mL	297.42
K_01_	1/h	4.51
*T*_lag_	h	0.18
*Distribution phase*		
K_12_	1/h	2.69
K_21_	1/h	0.15
*Elimination phase*		
K_10_	1/h	1.77

**Figure 7:**
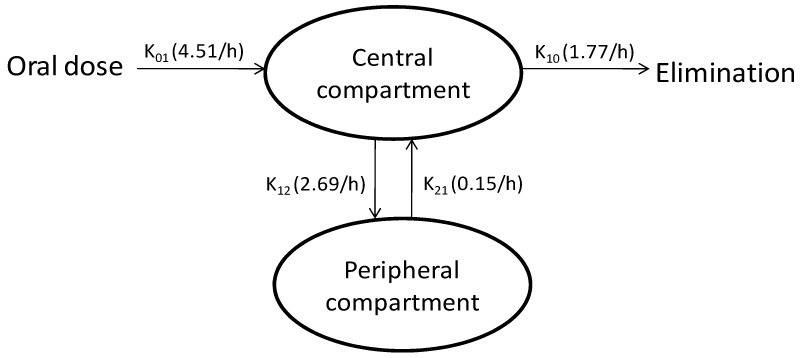
Pharmacokinetic model developed to describe the plasma concentration-time profile in human volunteers after oral dosing of CRM-LF.

A close evaluation of the obtained primary pharmacokinetic parameters provided valuable insight into the behavior of the CRM, after absorption from CRM-LF. CRM-LF showed a lag time (*T*_lag_) of 0.18 h (around 12 min). A high absorption rate constant (*K*_01_, 4.51/h) signifies that CRM-LF ensured rapid absorption of the CRM into the central compartment. A high value of *V*_D1_ (1421.6 L) suggested that CRM was extensively distributed in to tissues and/or bound to the tissue and/or cell organelles. It can also be seen that the distribution rate constant from central to peripheral compartment (*K*_12_, 2.69/h) is significantly higher than the rate constant from peripheral to central compartment (K_21_, 0.15/h) and overall elimination rate constant (*K*_10_, 1.77/h). Hence, low plasma concentrations observed for CRM after oral administration in various studies is not only due to poor absorption and rapid metabolism, but also due to the rapid and extensive distribution of the absorbed CRM into the peripheral compartment and its slower return into the central compartment (Figure 7). This interesting finding of the present study was also supported by a previous study on *in vitro* permeability of CRM done in our laboratory [9]. CRM demonstrated higher rate of absorption in comparison to the rate of desorption, when incubated in suspension of Caco-2 cells. At the end of a 2 h study, more than 20% CRM was ‘trapped’ inside the Caco-2 cells. Confocal laser scanning microscopy (CLSM) studies had also confirmed these observations. This trapping may be a result of interaction between –C=O group of CRM and –SH groups of groups of cysteine residues of different proteins.

Lopez-Lazaro *et al*. [21] had demonstrated that the two electrophilic α, β unsaturated “ketones” of CRM can bind covalently with the thiol (SH) groups of cysteine residues of different proteins. This interaction has also been held responsible for inducing the formation of topo II-DNA complexes by CRM [22,23]. Additionally, Barry *et al*. [24] had predicted that CRM interacts with the lipid headgroups in the plasma membrane and affects the functions of membrane proteins. This hypothesis is further strengthened by the fact that CRM is a highly lipophilic compound with a log *P* of 3.28, encouraging its accumulation in the tissues. It is possible that repeated oral dosing might accumulate CRM in the peripheral compartment and higher concentrations may be achieved in the tissues, as compared to plasma. 

These observations provide interesting insights into the pharmacokinetic-pharmacodynamic correlation of CRM. Several reports on pharmacodynamics of CRM were not able to detect plasma levels, even though there was positive evidence of therapeutic activity. This atypical behavior of CRM is in line with previously reported observations for azithromycin, a widely used macrolide antibiotic. Paolo *et al*. [25] studied the pharmacokinetics of azithromycin, which is characterized by rapid and extensive uptake within the intracellular and interstitial compartments of tissues, like lung tissues, though its plasma levels are low. This is the reason for extended half-life and long duration of action of azithromycin, despite of low plasma levels. It may therefore be postulated that CRM maintains therapeutically relevant levels in the peripheral tissues, though plasma levels might be well below the limit of detection of the employed analytical method. This pharmacokinetic aspect, as pertains to oral delivery of CRM, is being currently addressed by our research group by performing tissue distribution studies of CRM, after oral administration of CRM-LF. 

The clinical outcome for majority of drugs is predicted by estimating the plasma-based pharmacokinetics. However, this approach may not be appropriate for rapidly absorbed and distributed drugs like CRM and azithromycin. Additionally, drug concentration at target tissue will dictate the efficacy this can serve as a better surrogate of therapeutic activity of drug. 

## 4. Conclusions

CRM is rapidly absorbed after oral administration of CRM-LF and distributed rapidly into tissues, resulting in very low or undetectable plasma levels. Pharmacokinetic modeling of the plasma concentration-time profile after oral administration revealed that CRM-LF followed the two-compartment model with first order absorption, lag time and first order elimination. The study provides insight into the therapeutic efficacy of CRM despite undetectable levels in plasma. 

## References

[1] Ratnam D.V., Ankola D.D., Bhardwaj V., Sahana D.K., Ravi Kumar M.N.V. (2006). Role of antioxidants in prophylaxis and therapy: A pharmaceutical perspective. J. Control. Rel..

[2] De Toma A.S., Salamekh S., Ramamoorthy A., Lim M.H. (2012). Misfolded proteins in Alzheimer’s disease and type II diabetes. Chem. Soc. Rev..

[3] Popovych N., Brender J.R., Soong R., Vivekanandan S., Hartman K., Basrur V.,  Macdonald P.M., Ramamoorthy A. (2012). Site specific interaction of the polyphenol EGCG with the SEVI amyloid precursor peptide PAP(248−286). J. Phys. Chem..

[4] Huang R., Vivekanandan S., Brender J.R., Abe Y., Naito A., Ramamoorthy A. (2012). NMR characterization of monomeric and oligomeric conformations of human calcitonin and its interaction with EGCG. J. Mol. Biol..

[5] Aggarwal B.B., Sundaram C., Malani N., Ichikawa H. (2007). Curcumin: The Indian solid gold. Adv. Exp. Med. Biol..

[6] Aggarwal B.B., Harikumar K.B. (2009). Potential therapeutic effects of curcumin, the anti-inflammatory agent, against neurodegenerative, cardiovascular, pulmonary, metabolic, autoimmune and neoplastic diseases. Int. J. Biochem. Cell Biol..

[7] Wang Y.J., Pan M.H., Cheng A.L., Lin L.I., Ho Y.S., Hsieh C.Y., Lin J.K. (1997). Stability of curcumin in buffer solutions and characterization of its degradation products. J. Pharm. Biomed. Anal..

[8] Pan M.H., Huang T.M., Lin J.K. (1999). Biotransformation of curcumin through reduction and glucuronidation in mice. Drug Metab. Dispos..

[9] Wahlang B., Pawar Y.B., Bansal A.K. (2011). Identification of permeability-related hurdles in oral delivery of curcumin using the Caco-2 cell model. Eur. J. Pharm. Biopharm..

[10] Sharma R.A., Euden S.A., Platton S.L., Cooke D.N., Shafayat A., Hewitt H.R., Marczylo T.H., Morgan B., Hemingway D., Plummer S.M. (2004). Phase I clinical trial of oral curcumin: biomarkers of systemic activity and compliance. Clin. Cancer Res..

[11] Anand P., Kunnumakkara A.B., Newman R.A., Aggarwal B.B. (2007). Bioavailability of curcumin: Problems and promises. Mol. Pharm..

[12] Chattopadhyay I., Biswas K., Bandyopadhyay U., Banerjee R.K. (2004). Turmeric and curcumin: Biological actions and medicinal applications. Curr. Sci..

[13] Munjal B., Pawar Y.B., Patel S.B., Bansal A.K. (2011). Comparative oral bioavailability advantage from curcumin formulations. Drug Deliv. Transl. Res..

[14] Dhillon N., Aggarwal B.B., Newman R.A., Wolff R.A., Kunnumakkara A.B., Abbruzzese J.L., Ng C.S., Badmaev V., Kurzrock R. (2008). Phase II trial of curcumin in patients with advanced pancreatic cancer. Clin. Cancer Res..

[15] Sharma R.A., McLelland H.R., Hill K.A., Ireson C.R., Euden S.A., Manson M.M., Pirmohamed M., Marnett L.J., Gescher A.J., Steward W.P. (2001). Pharmacodynamic and pharmacokinetic study of oral Curcuma extract in patients with colorectal cancer. Clin. Cancer Res..

[16] Lao C.D., Ruffin M.T., Normolle D., Heath D.D., Murray S.I., Bailey J.M., Boggs M.E., Crowell J., Rock C.L., Brenner D.E. (2006). Dose escalation of a curcuminoid formulation. BMC Complement. Altern. Med..

[17] Antony B., Merina B., Iyer V.S., Judy N., Lennertz K., Joyal S. (2008). A pilot cross-over study to evaluate human oral bioavailability of BCM-95^®^ CG (BiocurcumaxTM), a novel bioenhanced preparation of curcumin. Ind. J. Pharm. Sci..

[18] Gota V.S., Maru G.B., Soni T.G., Gandhi T.R., Kochar N., Agarwal M.G. (2010). Safety and pharmacokinetics of a solid lipid curcumin particle formulation in osteosarcoma patients and healthy volunteers. J. Agric. Food Chem..

[19] Wahlang B., Kabra D., Pawar Y.B., Tikoo K., Bansal A.K. (2012). Contribution of formulation and excipients towards enhanced permeation of curcumin. Arzneimittelforschung.

[20] Pawar Y.B. (2011). Department of Pharmaceutics, NIPER, SAS Nagar, Punjab, India.

[21] Lopez-Lazaro M. (2008). Anticancer and carcinogenic properties of curcumin: Considerations for its clinical development as a cancer chemopreventive and chemotherapeutic agent. Mol. Nutr. Food Res..

[22] Martin-Cordero C., Lopez-Lazaro M., Galvez M., Ayuso M.J. (2003). Curcumin as a DNA topoisomerase II poison. J. Enz. Inhib. Med. Chem..

[23] Wang H., Mao Y., Chen A.Y., Zhou N., LaVoie E.J., Liu L.F. (2001). Stimulation of topoisomerase II-mediated DNA damage via a mechanism involving protein thiolation. Biochem..

[24] Barry J., Fritz M., Brender J.R., Smith P.E.S., Lee D.K., Ramamoorthy A. (2009). Determining the Effects of Lipophilic Drugs on Membrane Structure by Solid-State NMR Spectroscopy: The Case of the Antioxidant Curcumin. J. Am. Chem. Soc..

[25] Paolo A.D., Barbara C., Chella A., Angeletti C.A., Tacca M.D. (2002). Pharmacokinetics of azithromycin in lung tissue, bronchial washing, and plasma in patients given multiple oral doses of 500 and 1000 mg daily. Pharmacol. Res..

